# Platelet membrane-modified exosomes targeting plaques to activate autophagy in vascular smooth muscle cells for atherosclerotic therapy

**DOI:** 10.1007/s13346-025-01792-1

**Published:** 2025-01-28

**Authors:** Yu Jiang, Zhi-Yao Wei, Zhi-Feng Song, Miao Yu, Ji Huang, Hai-Yan Qian

**Affiliations:** 1 Center for Coronary Heart Disease, Department of Cardiology, National Center for Cardiovascular Diseases of China, State Key Laboratory of Cardiovascular Disease, Fu Wai Hospital, Chinese Academy of Medical Sciences and Peking Union Medical College, 167 Beilishi Rd, Beijing, 100037 China; 2 Center for Coronary Artery Disease, Division of Cardiology, Beijing Anzhen Hospital, Beijing Institute of Heart, Lung, and Blood Vessel Diseases, Capital Medical University, National Clinical Research Center for Cardiovascular Diseases, Beijing, China

**Keywords:** Atherosclerosis, Exosome, Targeting delivery, Vascular smooth muscle cells, Autophagy

## Abstract

Atherosclerosis is one of the leading causes of ischemic cardiovascular disease worldwide. Recent studies indicated that vascular smooth muscle cells (VSMCs) play an indispensable role in the progression of atherosclerosis. Exosomes derived from mesenchymal stem cells (MSCs) have demonstrated promising clinical applications in the treatment of atherosclerosis. However, there are still challenges and limitations persist in targeted therapy. This study aims to develop a bionic nano-delivery system by fusing platelet membranes with exosomes (MSC-Exo^P^) and explore the anti-atherosclerosis effect of MSC-Exo^P^ by improving the targeting efficiency and participating in regulating the pathophysiological processes associated with VSMCs. The morphology, particle size, stability, and fusion efficiency of MSC-Exo^P^ were assessed using transmission electron microscopy (TEM), nanoparticle tracking analysis (NTA), immunofluorescence staining, and Western blotting, respectively. MSC-Exo^P^ was administered intravenously into ApoE^−/−^ mice via the tail vein. In vivo, immunofluorescence staining was used to assess the targeting efficacy of MSC-Exo^P^. The ORO staining, H&E staining, Masson staining, aortic root immunofluorescence staining, and Western blot were utilized to evaluate the VSMC autophagy and anti-atherosclerosis effects of MSC-Exo^P^. In vitro, the autophagy activation of MSC-Exo^P^ on VSMCs was further assessed by immunofluorescence staining and Western blotting. The effects of MSC-Exo^P^ on VSMCs proliferation, migration, and foam cell formation were detected by EdU experiment, Transwell experiment, wound healing experiment, ORO staining, and BODIPY staining. The TEM revealed that MSC-Exo^P^ retained a ring nanostructure, which was similar to MSC-Exo in morphology. NTA analysis indicated the MSC-Exo^P^ exhibited a slight increase after cell membrane fusion. Besides, the stability analysis of exosomes and MSC-Exo^P^ resulted in no significant changes in particle size. Western blot analysis confirmed that MSC-Exo^P^ simultaneously expressed platelet-specific markers (GPVI, GPIbα, CD62P) and exosome-specific markers (CD81, TSG101, and Alix). In ApoE^−/−^ mice, the immunofluorescence of aorta and its roots was significantly enhanced after injection of DiI-labeled MSC-Exo^P^, indicating enhanced targeting of MSC-Exo to atherosclerotic plaques by platelets. In vivo experiments demonstrated that MSC-Exo^P^ could significantly suppress the progression of atherosclerosis and reduce the area of atherosclerotic plaques by reducing lipid deposition and necrotic nucleus area and increasing collagen content. In vitro experiments further revealed that the uptake of MSC-Exo^P^ by foam cells significantly increased, and their proliferation, migration, and foam formation were inhibited by autophagy activation. This study demonstrated successful fusion of platelet membranes with exosomes derived from MSCs. MSC-Exo^P^ could significantly improve the targeting efficiency of atherosclerosis and play an anti-atherosclerosis effect by activating VSMC autophagy.

**Graphical Abstract:**

**Figure:**
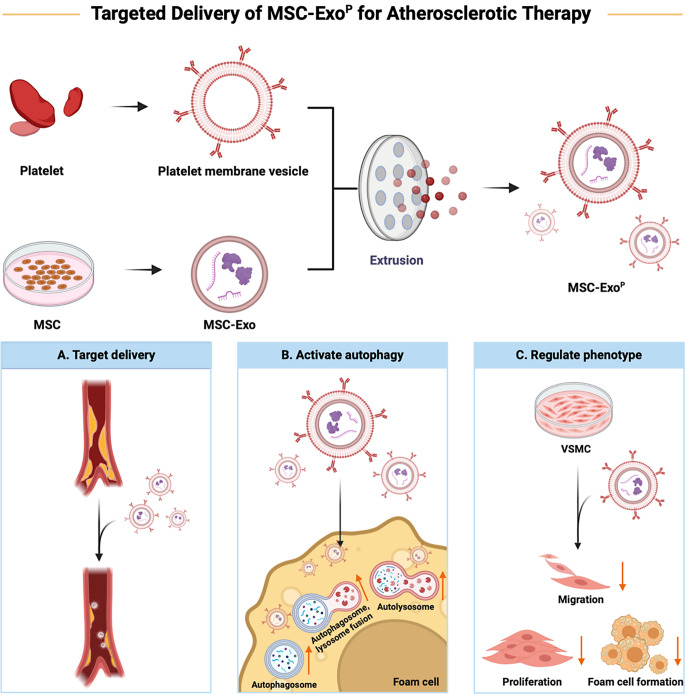
**Scheme 1** Graphical abstract of targeted delivery of MSC-ExoP to plaque for the treatment of atherosclerosis

## Introduction

Atherosclerosis, characterized by the formation of lipid pools and fibrous caps, is a chronic progressive inflammatory disease, which is one of the leading causes of mortality in cardiovascular diseases worldwide [1]. Traditional medical treatments mainly focus on alleviating symptoms rather than decreasing or reversing the underlying atherosclerotic plaque. Despite recent advances in our understanding of atherosclerosis, the lack of definitive clarity of the origin and function of many cells involved in atherosclerosis limited the development of effective therapies to prevent or treat the disease [2]. Recently, the cell lineage tracer technique and single-cell epigenetic assay revealed that both the function and the number of vascular smooth muscle cells (VSMCs) in atherosclerotic plaques have been historically underestimated [3, 4]. Wang et al. demonstrated that VSMCs are the primary source of 30-70% of foam cells in atherosclerotic plaques, not from macrophages [5]. Stimulated by oxidized low-density lipoprotein (ox-LDL) and inflammatory factors, VSMCs could undergo proliferative and migratory responses, leading to the stenosis of vascular lumens and the formation of fibrotic caps [6–8]. Therefore, VSMCs are likely to be the main culprits responsible for the progression of atherosclerosis.

Recent advances in the field of stem cells and their exosomal derivatives have shown promising potential for stem cell-based therapy of atherosclerosis. Exosomes, a paracrine vesicle with abundant proteins, cytokines, lipids, and non-coding RNA [9], are critical mediators of cellular information communications and considered as ideal targets for impeding the progression of atherosclerosis [10]. Unlike traditional stem cell transplantation therapy, exosome-based therapy can avoid the mechanical injury caused by transplantation and reduce the possibility of tumorigenesis [11]. Despite the potential effectiveness of exosome-based therapy, the application still faces several challenges, which mainly focus on low production in conventional cultivated conditions and poor delivery efficiency after intravenous injections [12]. To improve the targeting ability of exosomes, platelet-related membrane systems have uncovered their unique potentialities in the treatment of atherosclerosis [13]. Owing to the natural targeting ability with no thrombogenic risk, platelet membrane modified nanomedicine has been extensively studied in atherosclerosis [14, 15]. Inspired by the platelet membrane modification technology, infusing the platelet membrane modified exosomes derived from mesenchymal stem cells (MSC-Exo) may potentially enhance the targeting efficiency and therapeutic effect in atherosclerosis.

Autophagy is a normal physiological process that maintains the homeostasis of the internal environment and is closely associated with various pathological and physiological processes of VSMCs [16–19]. The autophagy process begins with the formation of phagophores, which can engulf cytoplasm, improperly folded proteins, and dysfunctional cellular organelles. Subsequently, the phagophores merge with lysosomes to decompose the inclusions and complete the recycling of matter and energy [20]. Pi et al. [18] found that inhibition of autophagy could reduce cholesterol efflux from VSMC-derived foam cells and eventually promote atherosclerotic progression. Besides, knocking out autophagy gene Atg7 could induce impaired autophagy in VSMCs, which promoted VSMC phenotype switching and increased the instability of plaque and the risk of plaque rupture [21, 22]. Rapamycin, an activator of autophagy, has been utilized on drug-eluting stents to prevent coronary artery restenosis and inhibit the proliferation and migration of VSMCs [23]. Moreover, evidence suggests that activation of autophagy could promote VSMC survival, inhibit cellular senescence, and decrease necrotic core area [24]. Targeting autophagy of VSMCs could be an integral target in regressing the onset and progression of atherosclerosis.

**Scheme 1:**
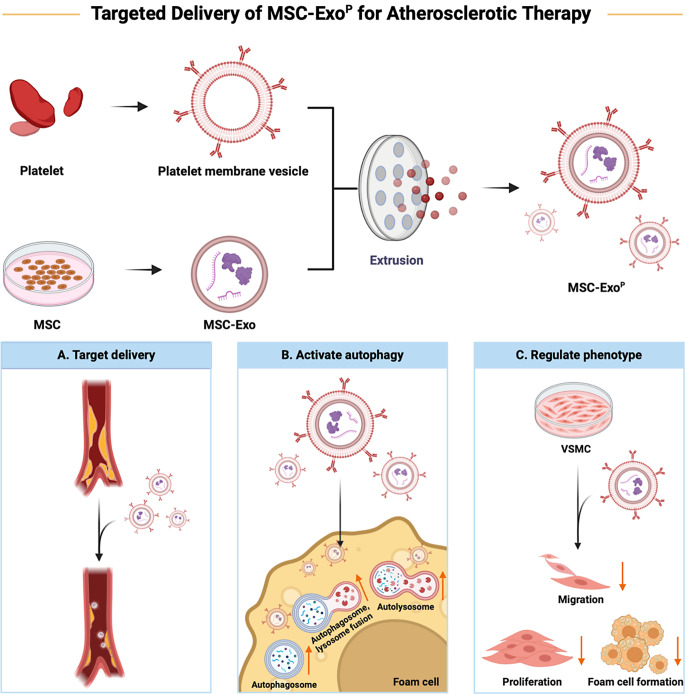
Graphical abstract of targeted delivery of MSC-ExoP to plaque for the treatment of atherosclerosis

Herein, we propose to improve the targeting ability and therapeutic effect of MSC-Exo on atherosclerosis by developing a hybrid exosome with platelet membrane (MSC-Exo^P^) (Scheme 1). Our objective is to determine whether MSC-Exo^P^ shows a superior anti-atherogenic effect compared to MSC-Exo alone. We plan to evaluate the MSC-Exo^P^ targeting ability and investigate the detailed mechanisms of MSC-Exo^P^ on atherosclerosis, including suppressing VSMC proliferation, migration, and foam cell formation through activating autophagy, both in vitro and in ApoE^−/−^ mice model. In this work, our study sheds light on the potential of MSC-Exo^P^ as a targeting therapy for atherosclerosis.

## Materials and methods

### Cell culture

Bone marrow MSCs were isolated as previously described [25]. Briefly, the tibias and femurs were dissected, and total bone marrow cells were flushed using culture media. The cells were incubated with Iscove’s Modified Dulbecco’s Medium (IMDM, Gibco, 12440053) supplemented with 10% fetal bovine serum (Sigma-Aldrich, F8318) and 1% penicillin-streptomycin (Gibco, 15140122). MSCs at P3-P5 were used for the subsequent experiments. Mouse aortic VSMCs were obtained as previously described [18]. Cells were cultured in Dulbecco’s Modified Eagle Medium/Nutrient Mixture F-12 (DMEM/F-12, Sigma-Aldrich, D8437) containing 10% fetal bovine serum, and 1% penicillin-streptomycin. VSMCs at P6-P8 were used for the following experiments. All cells were incubated in a humidified chamber at 37 °C in 5% CO_2_ atmosphere.

### Isolation of extracellular vesicles

To isolate exosomes from MSCs, MSCs were seeded in plates and cultured in serum-free medium for another 48 h. The supernatants were collected and centrifuged through several steps at 4 °C (300 g for 10 min; 2000 g for 20 min) to remove cells and cell debris. The supernatants were then centrifuged at 13,500 g for 30 min to eliminate large extracellular vesicles. Finally, the sample was ultracentrifuged twice at 120,000 g for 70 min using an Optima L-100XP Ultracentrifuge (Beckman, USA) to obtain exosome pellets. The exosome pellets were then washed in appropriate volume of phosphate-buffered saline (PBS) and stored at − 80 °C for use in experiments.

### Platelet isolation and platelet membrane extraction

The platelets were collected from platelet rich plasma (PRP) as described previously [15]. In detail, fresh human type O blood was collected using anti-coagulant tubes to prevent platelet activation. The whole blood was centrifuged (100 g, 20 min) at room temperature to separate and extract the supernatant PRP. The resulting PRP was then centrifuged again (100 g, 20 min) to remove remaining blood cells. Then PRP was mixed with PBS containing 1mM Ethylene Diamine Tetraacetic Acid (EDTA, Solarbio, E8030) and 2 mM of prostaglandin E1 (PGE1, Sigma-Aldrich, 900100P) to prevent platelet activation. Platelets were obtained by centrifugation (800 g, 20 min) at room temperature and then resuspended in PBS containing 1 mM EDTA and protease inhibitor. The platelet membranes were obtained after repeated freeze-thaw process and pelleted by centrifugation at 4000 g for 3 min. Then the platelet membranes were sonicated for 5 min in Ultrasonic Cell Crusher VCX130 (Sonics, USA) at a frequency of 42 kHz and a power of 100 W, and extruded sequentially through 400 nm and 200 nm polycarbonate porous membranes (Sigma-Aldrich, 610007 and 610006) 10 times each in the Avanti mini extruder (Sigma-Aldrich, USA).

### MSC-Exo^P^ preparation and characterization

The protein content of exosomes and platelet membrane vesicles was measured using a BCA protein assay. MSC-Exo and platelet membrane vesicles were mixed at an equal protein content ratio in the presence of 5% polyethylene glycol (PEG, Solarbio, P8040). Then the mixture was extruded 10 times each through 400 nm and 200 nm polycarbonate porous membranes. The size distribution, morphology, and key membrane proteins of platelet membrane vesicles, MSC-Exo and MSC-Exo^P^ were measured by nanoparticle tracking analysis (NTA), transmission electron microscopy (TEM), and Western blot, respectively. The stability of MSC-Exo and MSC-Exo^P^ was assessed following incubation with PBS and 10% FBS at 37 °C. Then, particle size measurements were conducted at different time points using NTA.

### Membrane fusion test

To verify the membrane colocalization, the platelet membranes (PM) were labeled with DiO (Invitrogen, V22886, Ex/Em = 484/501 nm) for 30 min, and MSC-Exo were labeled with DiI (Invitrogen, V22888, Ex/Em = 553/570 nm) for the same period. The remaining free dyes were removed by ultracentrifugation at 120 000 g for 90 min. Two samples were extruded or not extruded through 400 nm and 200 nm polycarbonate porous membranes and then observed under laser confocal microscopy (Leica SP8) [26]. The samples were dispersed in glycerol to decrease their mobility and obtain stable images.

### Western blot analysis

The protein levels of MSC-Exo, MSC-Exo^P^, PM, VSMCs, and aorta tissues were examined using western blot. The protein was extracted using RIPA lysis buffer (Beyotime, P0013B) with protease inhibitors (Beyotime, P1005). After centrifugation, the protein concentration was determined using a BCA protein assay before electrophoresed on 4–12% Bis-Tris Gels (Thermo Fisher Scientific, NP0322BOX). The protein was then electroblotted onto polyvinylidene difluoride (PVDF) membranes (Beyotime, FFP28) and blocked with 5% non-fat milk. The membranes were incubated with the following antibodies: GPVI (Abcam, ab129019), CD62P (Abcam, ab182135), CD42b (Abcam, ab134087), Alix (Abcam, ab275377), CD81 (Abcam, ab109201), CD73 (Abcam, ab175396), TSG-101 (Abcam, ab125011), LC3 (Abcam, ab192890), p62 (CST, 39749), PCNA (CST, 13110), and MMP-2 (Abcam, ab92536). The β-actin (Abcam, ab8226) was used as an internal control. Subsequently, the membranes were incubated with an HRP-conjugated antibody. Detection was performed using a Chemiluminescence imaging system (Tanon 5800 Multi, China).

### Cellular uptake assay

To evaluate the uptake efficiency by VSMCs, VSMCs were pre-stimulated with ox-LDL (Yiyuan Biotech, YB-002; 80 µg/mL) for 24 h and then cocultured with DiI-labelled MSC-Exo or MSC-Exo^P^ for 6 h. VSMCs were washed with PBS and stained with phalloidin (Abcam, ab176759, Ex/Em = 650/665 nm). The internalization of DiI-labelled MSC-Exo or MSC-Exo^P^ by VSMCs was observed under laser confocal microscopy.

### Histology and immunofluorescence staining

For histologic analysis, ORO, HE, and Masson staining were performed on frozen section (10 μm thick) of aortic roots. The sections were then observed by optical microscopy (Olympus, Japan). For immunofluorescence analysis, the frozen sections were incubated with antibodies against LC3 (Abcam, ab192890) and ACTA2 (Abcam, ab7817). For VSMCs, after being fixed with 4% paraformaldehyde (Leagene, DF0135) for 30 min, the VSMCs were then permeabilized, and incubated with antibodies against LC3 (ab192890). They were then detected by fluorescently labeled secondary antibodies, and the nucleus was stained with Hoechst (Sigma-Aldrich, 63493). The sections or VSMCs were observed under the Leica SP8.

### In vivo targeting to atherosclerotic plaque

To determine the in vivo targeting properties of MSC-Exo^P^, ApoE^−/−^ mice were injected with PBS, DiI-labeled MSC-Exo, or DiI‐labeled MSC-Exo^P^ via the tail vein, respectively. Then, the mice were sacrificed 6 h after intravenous injection. The fluorescence imaging of the arterial trees was imaged under Pannoramic SCAN (3DHistech, Hungary). For local distribution in the aortic roots, frozen sections were observed under laser confocal microscopy.

### Establishment of atherosclerosis model and treatment protocol

All animals were obtained from the Animal Department of Fuwai Hospital. The animal experiment was conducted under the approval and supervision of the Experimental Animals Ethics Committee of Fuwai Hospital (FW-2022-0027), and all procedures met the National Institutes of Health guidelines.

After 12 weeks of a western-type diet (Research diets, D12108C, 40 kcal% fat and 1.25% cholesterol) to induce atherosclerosis, ApoE^−/−^ mice were divided into four groups (*n* = 6): [1] control group [2], MSC-Exo group [3], MSC-Exo^P^ group [4], MSC-Exo^P^+3-MA group. The mice were subjected to various treatments twice a week via the tail vein for additional 4 weeks, while kept on a western-type diet during treatment. The control group, MSC-Exo group, MSC-Exo^P^ group, and MSC-Exo^P^+3-MA group were respectively injected with PBS, MSC-Exo (150 µg), MSC-Exo^P^ (150 µg Exo) and MSC-Exo^P^ (150 µg Exo). To analyze the influence of autophagy in vivo, MSC-Exo^P^+3-MA group was intraperitoneally injected with 3-MA (MCE, HY-19312, 3 mg/100 g) every other day, and saline was intraperitoneally administered as a control treatment.

### Atherosclerotic lesion analysis

For the quantification of atherosclerotic plaque lesions in aorta from the aortic root to the iliac bifurcation, the whole aortas were carefully peeled of perivascular fat and opened longitudinally. Then, the aortas were pinned enface and stained with ORO (Sigma-Aldrich, O0625). The specimen was photographed using optical microscopy and the ORO-positive areas were measured with Image J software.

### ORO staining

To obtain foam cells, VSMCs were stimulated with 80 µg/mL oxLDL for 24 h. VSMCs were subsequently fixed in 4% paraformaldehyde for 15 min and washed with PBS three times. The cells were then stained with ORO solution for 30 min at room temperature. After washing with 60% isopropanol, nucleus was stained with hematoxylin staining solution, and ORO staining was observed by optical microscopy and quantified by Image J software.

### BODIPY staining

For staining lipid droplets, the VSMCs or sections were stained with BODIPY 493/503 (Thermo Fisher Scientific, D3922) at 10 µM for 30 min at 37 °C in the dark. Finally, the samples were stained with Hoechst, washed with PBS, and then imaged using laser confocal microscopy. Quantification was performed using Image J software.

### Autophagic flux

VSMCs were transfected with Ad-mRFP-GFP-LC3B (Hanbio, HBAP210 0001) at a multiplicity of infection (MOI) of 50 in DMEM/F12 medium. After transfection, autophagic flux was detected by laser confocal microscopy. Both mRFP (red) and GFP (green) were detected in autophagosomes, present as yellow dots. The GFP (green) was sensitive to the acidic environment and subsequently quenched in autolysosomes, only mRFP (red) could be detected in autolysosomes. The number of yellow (mRFP^+^ GFP^+^) and red (mRFP^+^ GFP^−^) dots was analyzed using Image J software. At least 15 cells were analyzed for each population. Autophagic flux was evaluated by the number change of yellow (autophagosomes) and red (autolysosomes) dots.

### EdU staining assay

EdU staining (Beyotime Biotechnology, C0078) was used to analyze the VSMC proliferation. Briefly, VSMCs were cultivated at an appropriate number in a 12-well plate. After 24 h of the indicated treatment, each well was incubated with EdU (10 µM) for 2 h. The cells were fixed in PBS containing 4% paraformaldehyde for 15 min at room temperature. After washing with PBS, the cells were incubated with PBS containing 0.3% TritonX-100 (Leagene, R00280) for 10 min. Finally, the samples were stained with Hoechst, washed in PBS, and then imaged using laser confocal microscopy.

### Transwell assay and scratch wound healing assay

VSMCs were analyzed by transwell assay and scratch wound healing assay to measure the degree of migration. For transwell assay, the VSMCs were placed in the upper chambers (Sigma-Aldrich, 3422) with serum-free medium, and the indicated treatment was added in the lower chambers with complete medium. After 24 h incubation, migrated VSMCs were moved from the upper chamber to the lower chamber. The migrated cells were stained with crystal violet (0.2%) and photographed in 5 random fields using a microscope. For scratch wound healing assay, the VSMCs were plated into 6-well plates and grown to 90% confluence. The monolayer was scratched vertically with a sterile pipette tip, followed by washing with serum-free medium to remove detached cells. Then, the scratches were observed and photographed at 0 h and 24 h, respectively. The VSMC migration distance was calculated as follows: migration area (%) = (M_0_– M_n_)/M_0_ × 100, M_0_ = the initial scratch wound area, and M_n_ = the scratch wound area at 24 h of measurement.

### Statistical analysis

All data were expressed as mean ± SD. The results were analyzed using Student’s t-test or one-way ANOVA in GraphPad Prism 9 and a P-value < 0.05 was considered statistically significant.

## Result

### Fabrication and characterization of MSC-Exo^P^

**Fig. 1:**
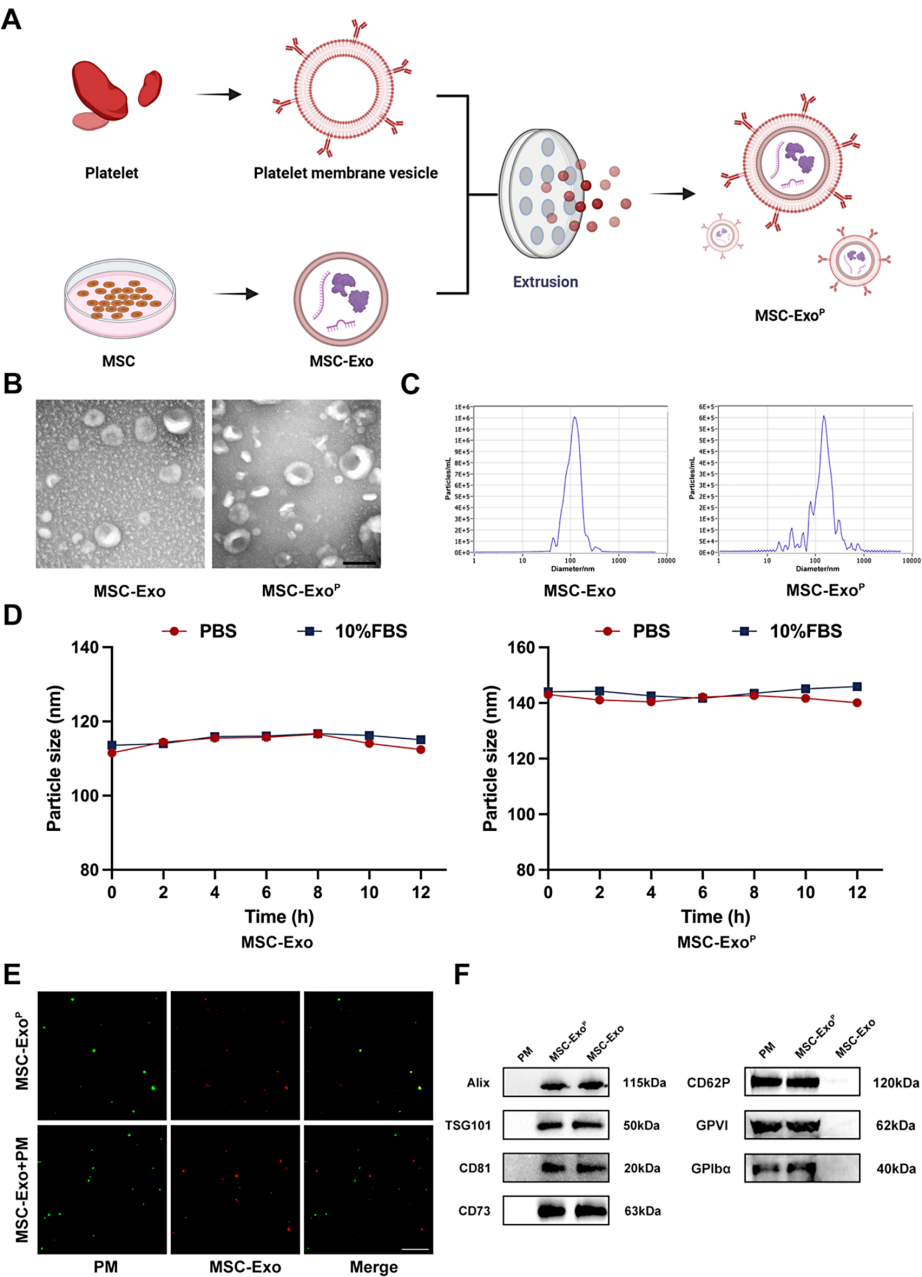
Fabrication and characterization of MSC-Exo^P^. (**A**) Schematic illustration showing a platelet-like delivery platform to plaques for the treatment on atherosclerosis. (**B**) Representative TEM images and (**C**) NTA size distribution of MSC-Exo and MSC-Exo^P^. Scale bar = 100 nm. (**D**) The particle sizes of exosome and MSC-Exo^P^ after incubation with PBS or 10% FBS. (**E**) Confocal fluorescence images of MSC-Exo^P^ (yellow) or a simple mixture of PM (green) and MSC-Exo (red). Scale bar = 20 μm. (**F**) Western blot analysis of CD81, TSG101, Alix and CD73 expression or GPVI, GPIbα and CD62P expression in platelet membrane (PM), MSC-Exo and MSC-Exo^P^

We developed a platelet-like delivery platform by fusing natural platelet membranes to the surface of MSC-derived exosomes for targeting therapy of atherosclerosis (Fig. 1A). Compared to platelet membrane vesicles and exosomes, most MSC-Exo^P^ were well coated with platelet membranes and showed core-shell structures in transmission electron microscope (TEM) images (Fig. 1B). The data revealed that the morphology of MSC-Exo^P^ remained unchanged after membrane coating modification. Nanoparticle Tracking Analysis (NTA) data showed the MSC-Exo^P^ exhibited a slight increase in size from 111.5 nm (MSC-Exo) to 143.0 nm (MSC-Exo^P^) after cell membrane fusion (Fig. 1C). Besides, the stability analysis of exosomes and MSC-Exo^P^ revealed that incubation in PBS or 10% serum for 12 h resulted in no significant changes in particle size (Fig. 1D). To further confirm the successful fusion of platelet membranes and exosomes, we labeled MSC-Exo with DiI lipophilic dye and labeled platelet membranes with DiO lipophilic dye to observe fluorescence colocalization relationship by confocal microscopy. As illustrated in Fig. 1E, the MSC-Exo^P^ group showed good colocalization, and no colocalization was shown in no extrusion group. Western blot analysis indicated that both the important platelet-specific markers (GPVI, GPIbα, CD62P) and exosome-specific markers (CD81, TSG101, and Alix) were well presented on MSC-Exo^P^. In addition to the specific markers mentioned above, MSC-Exo and MSC-Exo^P^ also expressed MSC surface marker CD73 (Fig. 1F). These data served as evidence that the process of fusion of exosomes and platelet membranes was successful, leading to the co-expression of membrane surface proteins from the two sources.

### Higher targeting ability and cellular uptake

**Fig. 2:**
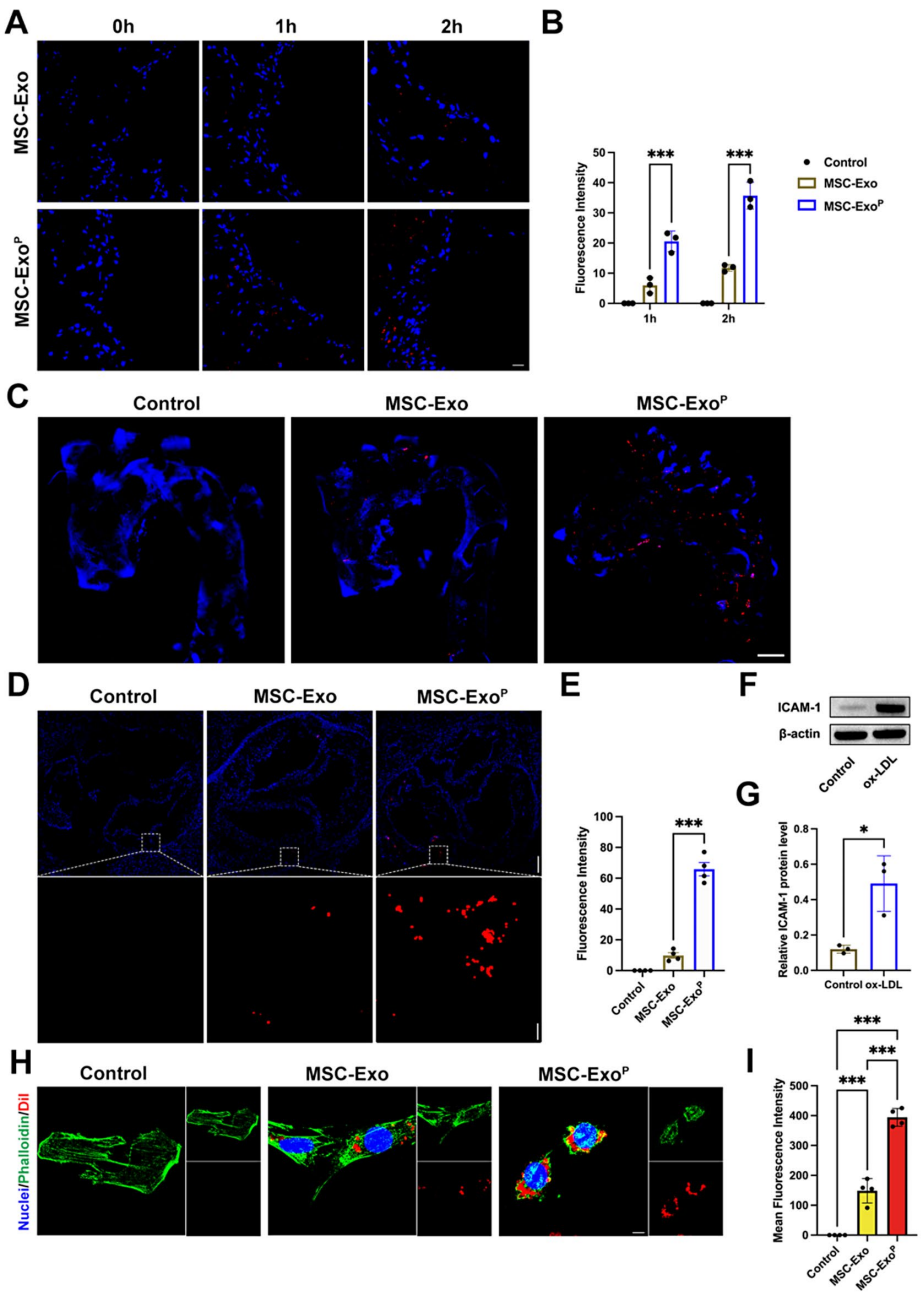
Targeting ability and cellular uptake. (**A**) Representative fluorescence images and (**B**) quantification of fluorescence intensity of isolated aortas at 1 h and 2 h after incubating with PBS, DiI-labeled MSC-Exo or MSC-Exo^P^ in vitro (red: MSC-Exo or MSC-Exo^P^, blue: nuclei). Scale bar = 20 μm. *n* = 3. (**C**) Fluorescence images of arterial trees after injecting with PBS, DiI-labeled MSC-Exo or MSC-Exo^P^ in vivo (red: MSC-Exo or MSC-Exo^P^, blue: nuclei). Scale bar = 1 mm. (**D**) Representative fluorescence images and (**E**) quantification of fluorescence intensity of aortic roots in vivo (red: MSC-Exo or MSC-Exo^P^, blue: nuclei). Scale bar = 150 μm and scale bar = 15 μm (Enlarge). *n* = 4. (**F**) Western blot analysis and (**G**) quantification of ICAM-1 protein level in VSMCs after treatment with ox-LDL. *n* = 3. (**H**) Representative immunofluorescence images and (**I**) quantitative analysis of DiI-labeled MSC-Exo or MSC-Exo^P^ uptake by VSMCs (red: MSC-Exo or MSC-Exo^P^, green: phalloidin, blue: nuclei). Scale bar = 5 μm. *n* = 4. All data are presented as mean ± SD (*P < 0.05, ***P < 0.001)

To assess the binding ability of MSC-Exo^P^ in vitro, the atherosclerotic aortas derived from mice were incubated with DiI-labeled MSC-Exo or MSC-Exo^P^. The results confirmed that MSC-Exo^P^ group displayed a 3.0-fold greater fluorescence intensity than MSC-Exo group after incubation for 1–2 h (*P* < 0.001), with no fluorescence in control group, suggesting that MSC-Exo^P^ had a superior targeting ability and accumulation in atherosclerotic plaques (Fig. 2A and B). To further assess the targeting ability of MSC-Exo^P^ in vivo, we observed the fluorescent intensity of intact and denuded aorta samples after intravenously injecting DiI-labeled MSC-Exo or MSC-Exo^P^. Our results demonstrated that the MSC-Exo^P^ group exhibited significantly higher fluorescence intensity than MSC-Exo group (Fig. 2C). Consistent with this result, the fluorescence intensity in aorta roots was observed using a confocal microscope. The higher fluorescence intensity in MSC-Exo^P^ group confirmed that the MSC-Exo^P^ had stronger ability to target atherosclerotic plaques compared with MSC-Exo (*P* < 0.001) (Fig. 2D and E). Then, we evaluated the cellular uptake efficiency of MSC-Exo^P^ by VSMCs. The VSMCs were stimulated with ox-LDL (80 µg/mL), and the expression of intracellular adhesion molecule-1 (ICAM-1) by VSMCs increased significantly (Fig. 2F and G), which greatly enhanced the ability of platelets to bind VSMCs. Following ox-LDL stimulation, the VSMCs were incubated with DiI-labeled MSC-Exo or MSC-Exo^P^ for a duration of 12 h. As showed in Fig. 2H and I, we observed a significant stronger red fluorescence intensity in the cytoplasm of MSC-Exo^P^ treated VSMCs than the MSC-Exo treated VSMCs (MSC-Exo 148.41 ± 35.30 versus MSC-Exo^P^ 394.24 ± 25.87; *P* < 0.001), indicating that platelet membrane fusion could potentially enhance the uptake efficiency by VSMCs.

### Alleviating the progression of atherosclerosis

**Fig. 3:**
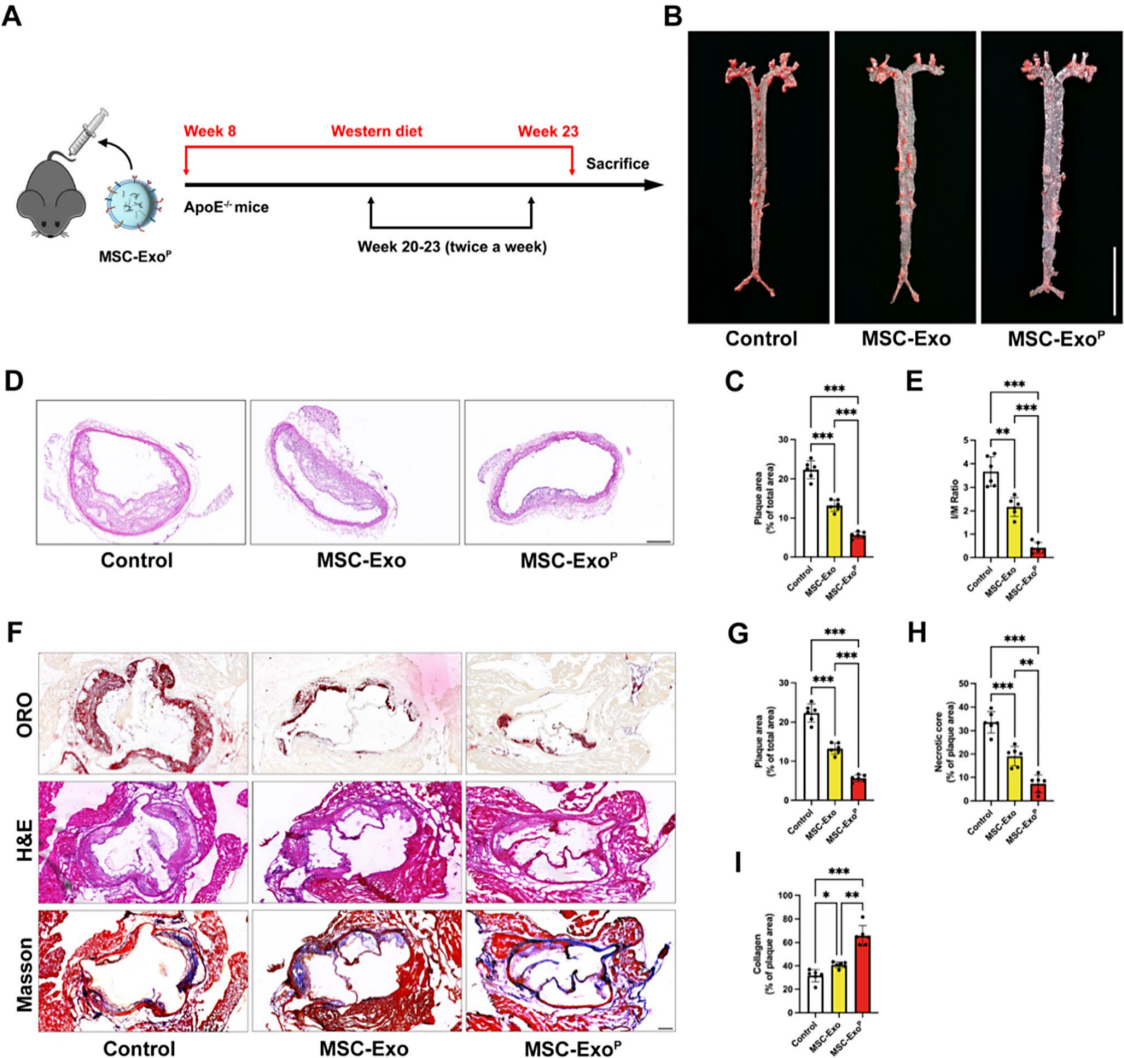
Regression of atherosclerosis in vivo. (**A**) Schematic representation illustrating of atherosclerosis model establishment and treatment protocol. (**B**) Representative images of en face ORO-stained total aortas and (**C**) quantitative analysis of plaque area after treatment with PBS, MSC-Exo and MSC-Exo^P^. Scale bar = 10 mm. *n* = 6. (**D**) Representative H&E images of the left common carotid artery after treatment with PBS, MSC-Exo and MSC-Exo^P^. Scale bar = 200 μm. *n* = 6. (**E**) Quantitative analysis of lesion area over the medial common area (intimal/medial area ratio) artery. (**F**) Pathological detections on cross sections from aortic roots by Oil Red, H&E and Masson after various treatments. Scale bar = 200 μm. *n* = 6. Quantitative analysis on (**G**) plaque area, (**H**) necrotic cores and (**I**) collagen content. All data are presented as mean ± SD (*P < 0.05, **P < 0.01, ***P < 0.001)

The in vivo anti-atherosclerosis effect was evaluated after 4-weeks treatment of MSC-Exo^P^ on ApoE^−/−^ mice (Fig. 3A). After staining the lesion areas with ORO, the images of entire arterial en face staining were exhibited in Fig. 3B. Further quantification based on ORO showed that treatment with MSC-Exo^P^ showed the strongest inhibition of plaque development verified by minimum plaque area (5.62 ± 0.83%) compared with MSC-Exo group (13.19 ± 1.42%, *P* < 0.001) and control group (22.32 ± 2.29%, *P* < 0.001) (Fig. 3C). Besides, the intimal/medial area ratio in the left common carotid artery as assessed by morphometry analysis was lower in MSC-Exo^P^ group than MSC-Exo^P^ group (*P* < 0.001) or control group (*P* < 0.001) (Fig. 3D and E). In line with what we observed above, ORO-stained cross-sections of aortic roots showed the similar results. The MSC-Exo^P^ group caused smaller lipid deposition and significantly reduced plaque lesions of aortic roots compared with any other group (*P* < 0.001) (Fig. 3F and G). Then, the cross-sections of aortic roots were stained with Hematoxylin-eosin (HE), and the results from Fig. 3F and H showed that the size of necrotic core area was remarkably decreased in MSC-Exo^P^ group (7.28 ± 3.71%) than MSC-Exo group (19.00 ± 4.18%, *P* < 0.01) or control group (33.59 ± 4.54%, *P* < 0.001). The Masson’s trichrome staining showed that the content of collagen in MSC-Exo^P^ group was 1.61-fold that of MSC-Exo group (*P* < 0.05) and 2.08-fold that of control group (*P* < 0.001) (Fig. 3F and I). Taken together, these results suggested that targeting delivery of MSC-Exo by platelet membrane vesicles could remarkably attenuate atherosclerotic development, further supporting the potential of MSC-Exo^P^ as an effective treatment option for atherosclerosis.

### Activation of VSMC autophagy in vitro

**Fig. 4:**
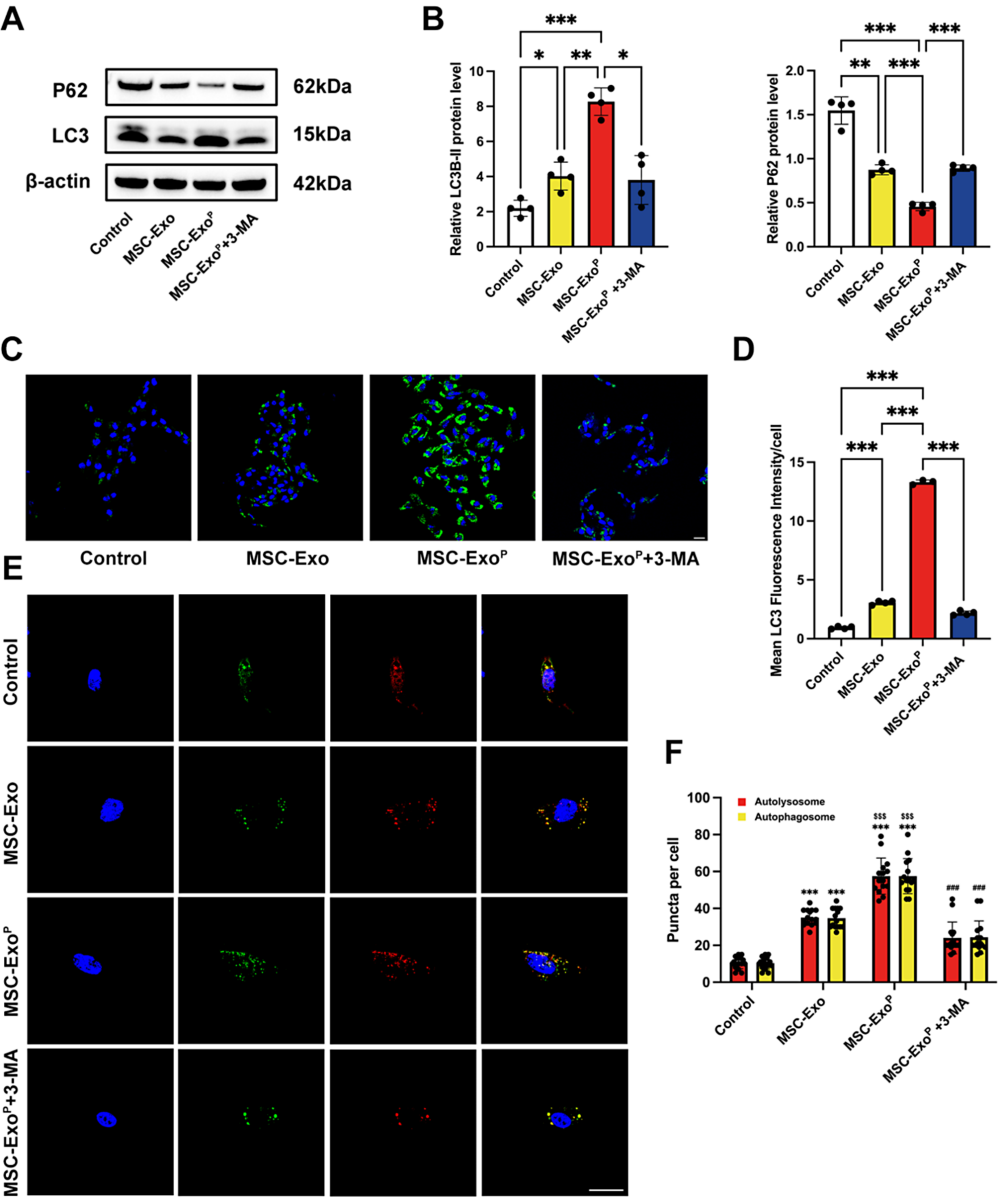
Activation of VSMC autophagy in vitro. (**A**) Western blot analysis and (**B**) quantification of LC3-II/ILC3-I and p62 after various treatments in vitro. *n* = 4. (**C**) Representative fluorescence images of LC3 staining in VSMCs after various treatments in vitro (green: LC3, blue: nuclei). Scale bar = 20 μm. *n* = 3–4. (**D**) Quantification of LC3 fluorescence intensity in VSMCs. (**E**) Representative fluorescence images for VSMCs transfected with ad-mRFP-GFP-LC3B and then treated with PBS, MSC-Exo, MSC-Exo^P^ or MSC-Exo^P^+3-MA (yellow: autophagosome dots, red: autolysosome dots, blue: nuclei). Scale bar = 5 μm. (**F**) Quantification of the number of autophagosome dots and autolysosome dots. *n* = 15. All data are presented as mean ± SD (For A-D, **P < 0.01, ***P < 0.001. For E-F, ***P < 0.001 compared with control group; ^**###**^*P* < 0.001 compared with MSC-Exo^P^ group; ^**$$$**^*P* < 0.001 compared with MSC-Exo group)

Previous studies demonstrated that the engagement of VSMC autophagy was associated with the progression of atherosclerosis [16, 17]. To ascertain whether MSC-Exo^P^ could regulate autophagy in VSMCs, we prestimulated VSMCs with ox-LDL and subsequently incubated with MSC-Exo or MSC-Exo^P^, respectively. We first detected the expression of LC3-II/LC3-I ratio, which was generally used for the detection of autophagic activity. The MSC-Exo^P^ group resulted in an overall increase in the level of LC3-II/LC3-I ratio compared with MSC-Exo group (*P* < 0.01) and control group (*P* < 0.001). Inhibition of autophagy was accompanied by the accumulation of p62 protein, we then examined p62 protein level, as expected, the MSC-Exo^P^ group displayed a lower level of p62 protein, and both the increase in LC3-II/LC3-I ratio and the decrease in p62 level were reversed by 3-MA (an autophagy inhibitor) treatment (both *P* < 0.05) (Fig. 4A and B). Additionally, we stained for LC3 in VSMCs treated with ox-LDL, and the fluorescence intensity of LC3 was stronger in MSC-Exo^P^ group compared with MSC-Exo group (*P* < 0.001) and control group (*P* < 0.001), while decreased after pretreatment with 3-MA compared with MSC-Exo^P^ treatment alone (Fig. 4C and D). Furthermore, we labeled LC3 and detected autophagy flux by using a double-labeled adenovirus that emited mRFP (red) and GFP (green). Generally, mRFP^+^ GFP^+^ LC3 pots (yellow dots) represented the presence of autophagosomes, whereas mRFP^+^ GFP^−^ dots (red dots) indicated the formation of autolysosomes because GFP was sensitive to the acidic environment. Figure 4E and F showed that the number of yellow and red dots significantly increased in MSC-Exo^P^ group, compared with MSC-Exo group (*P* < 0.001) and control group (*P* < 0.001). Moreover, the presence of the autophagy inhibitor 3-MA could decrease the number of yellow and red dots, corresponding to block the MSC-Exo^P^-induced autophagy flux. Collectively, these data revealed that the MSC-Exo^P^ group was more effective in activating VSMC autophagy compared to both MSC-Exo group and control group.

### Inhibition of VSMC proliferation and migration by enhancing autophagy

**Fig. 5:**
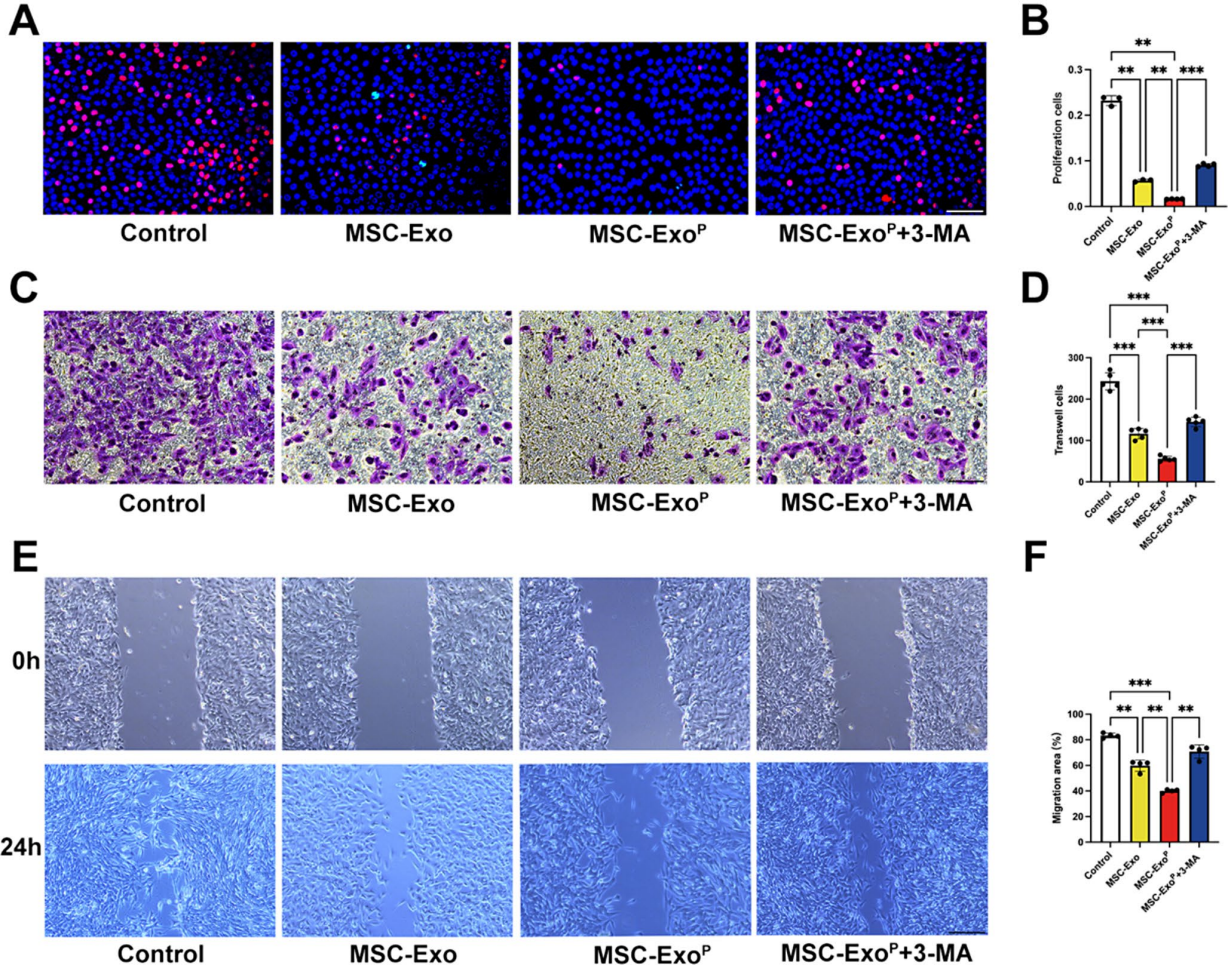
Inhibition of VSMC proliferation and migration in vitro. (**A**) Representative fluorescence images and (**B**) quantification of the EdU assay after treatment with PBS, MSC-Exo, MSC-Exo^P^ or MSC-Exo^P^+3-MA (red: EdU, blue: nuclei). Scale bar = 100 μm. *n* = 3–4. (**C**) Representative images of transwell assay after various treatments in vitro. Scale bar = 100 μm. *n* = 5. (**D**) Quantification of the number of migrated VSMCs. (**E**) Representative images of wound-healing assay and (**F**) quantification of migrated VSMCs at 0 and 24 h after different treatments in vitro. Scale bar = 200 μm. *n* = 4. All data are presented as mean ± SD (**P < 0.01, ***P < 0.001)

Excessive concentrations of ox-LDL stimulated the VSMC proliferation and migration, which eventually led to the progression of atherosclerosis [6]. In order to detect whether MSC-Exo^P^ could inhibit the proliferation and migration of VSMCs by regulating autophagy, we pretreated VSMCs with ox-LDL and then in combination with MSC-Exo or MSC-Exo^P^. After MSC-Exo^P^ treatment, the fluorescence signal in EdU staining significantly decreased in VSMCs compared with MSC-Exo group (*P* < 0.01) and control group (*P* < 0.01) (Fig. 5A and B). Besides, the transwell assay demonstrated that the MSC-Exo^P^ treatment resulted in greater suppression of VSMC migration, and these results were further validated in the wound-healing assay, which showed that MSC-Exo^P^ treatment had the smallest migration area (40.01 ± 1.11%) compared with MSC-Exo group (59.80 ± 3.79%; *P* < 0.01) and control group (83.35 ± 1.61%; *P* < 0.001) (Fig. 5C-F). To further explore the role of autophagy in VSMC proliferation and migration, we incubated MSC-Exo^P^ group with 3-MA. As expected, the inhibitory effect of MSC-Exo^P^ on VSMC proliferation could be reversed by the treatment of 3-MA (7.42% increase, *P* < 0.001) (Fig. 5A and B). Compared with MSC-Exo^P^ group, the MSC-Exo^P^+3-MA group showed increased migratory ability in the transwell assay (*P* < 0.001) and scratch wound assay (70.77 ± 4.51%; *P* < 0.01) (Fig. 5C-F). Overall, these data demonstrated that MSC-Exo^P^ could suppress VSMC proliferation and migration by activating autophagy in vitro, which was better than MSC-Exo.

### Inhibition of VSMC-derived foam cell formation by inducing autophagy

**Fig. 6:**
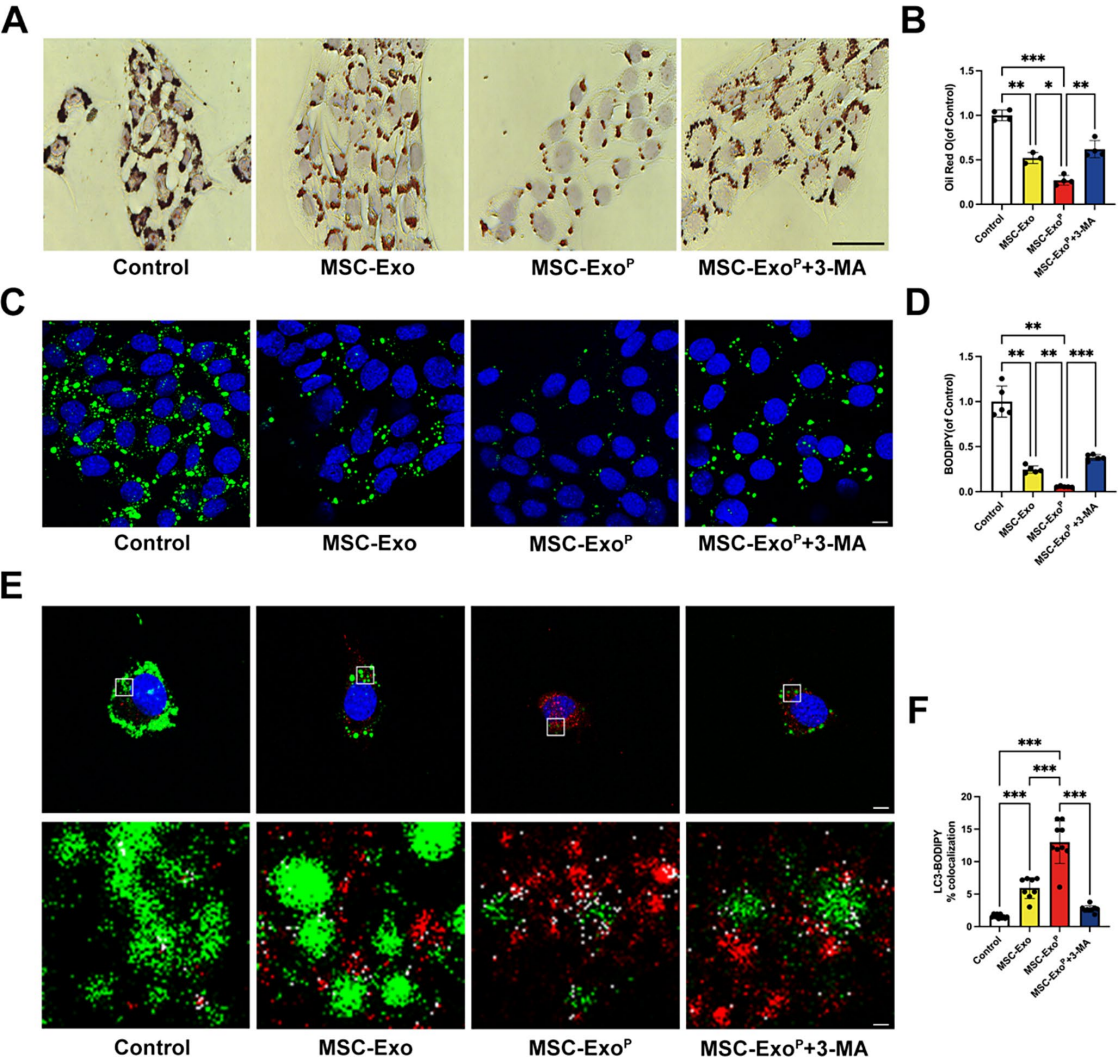
Inhibition of VSMC-derived foam cell formation in vitro. (**A**) Representative ORO images of ox-LDL-loaded VSMCs after treatment with PBS, MSC-Exo, MSC-Exo^P^ or MSC-Exo^P^+3-MA in vitro. Scale bar = 50 μm. *n* = 3–4. (**B**) Quantification of ORO area in VSMCs. (**C**) Representative confocal images and (**D**) quantification of BODIPY-positive area after different treatments in vitro (green: BODIPY, blue: nuclei). Scale bar = 10 μm. *n* = 5. (**E**) Representative confocal images of LC3-BODIPY in VSMCs after treatment with PBS, MSC-Exo, MSC-Exo^P^ or MSC-Exo^P^+3-MA in vitro. The magnified locations in the second row indicated colocalized pixels (red: LC3, green: BODIPY, white: colocalized pixels, blue: nuclei) Scale bar = 5 μm and scale bar = 500 nm (Enlarge). (**F**) Quantification of the percent colocalization between LC3 and BODIPY in VSMCs. *n* = 8–10. All data are presented as mean ± SD (*P < 0.05, **P < 0.01, ***P < 0.001)

Because VSMC-derived foam cells, the primary source of 30-70% of foam cells in atherosclerotic plaques, not from macrophages [5], have been proven to be indispensable in the process of atherosclerosis. We investigated whether MSC-Exo^P^ was involved in regulating VSMC-derived foam cell formation in vitro. We treated oxLDL-stimulated VSMCs with MSC-Exo or MSC-Exo^P^. We visualized cellular lipid accumulation by ORO or fluorescent BODIPY (an indicator for neutral fat deposition) staining, and the positive area was quantified. Depicted in Fig. 6A-D, we observed a remarkable reduction of lipid droplets stained in MSC-Exo^P^ group by ORO or BODIPY staining, compared with MSC-Exo group (*P* < 0.05) and control group (*P* < 0.01). In contrast, compared with MSC-Exo^P^ group, we found reversed effects on lipid droplets area after treatment of 3-MA both in ORO (*P* < 0.01) and BODIPY staining (*P* < 0.001), indicating that inhibition of autophagy increased VSMC-derived foam cell formation. Moreover, consistent with previous results, the colocalization analysis indicated that the colocalization of LC3-BODIPY increased when VSMCs were treated with MSC-Exo^P^ compared with MSC-Exo group (MSC-Exo^P^ 12.98 ± 3.07% versus MSC-Exo 5.93 ± 1.51%; *P* < 0.001), and treatment of 3-MA could abolish the increasing colocalization of LC3-BODIPY (2.70 ± 0.47%; *P* < 0.001) (Fig. 6E and F). These results indicated that the MSC-Exo^P^ further inhibited cellular lipid accumulation and foam cell formation in vitro via stimulating VSMC autophagy.

### In vivo selective activation of VSMC autophagy

**Fig. 7:**
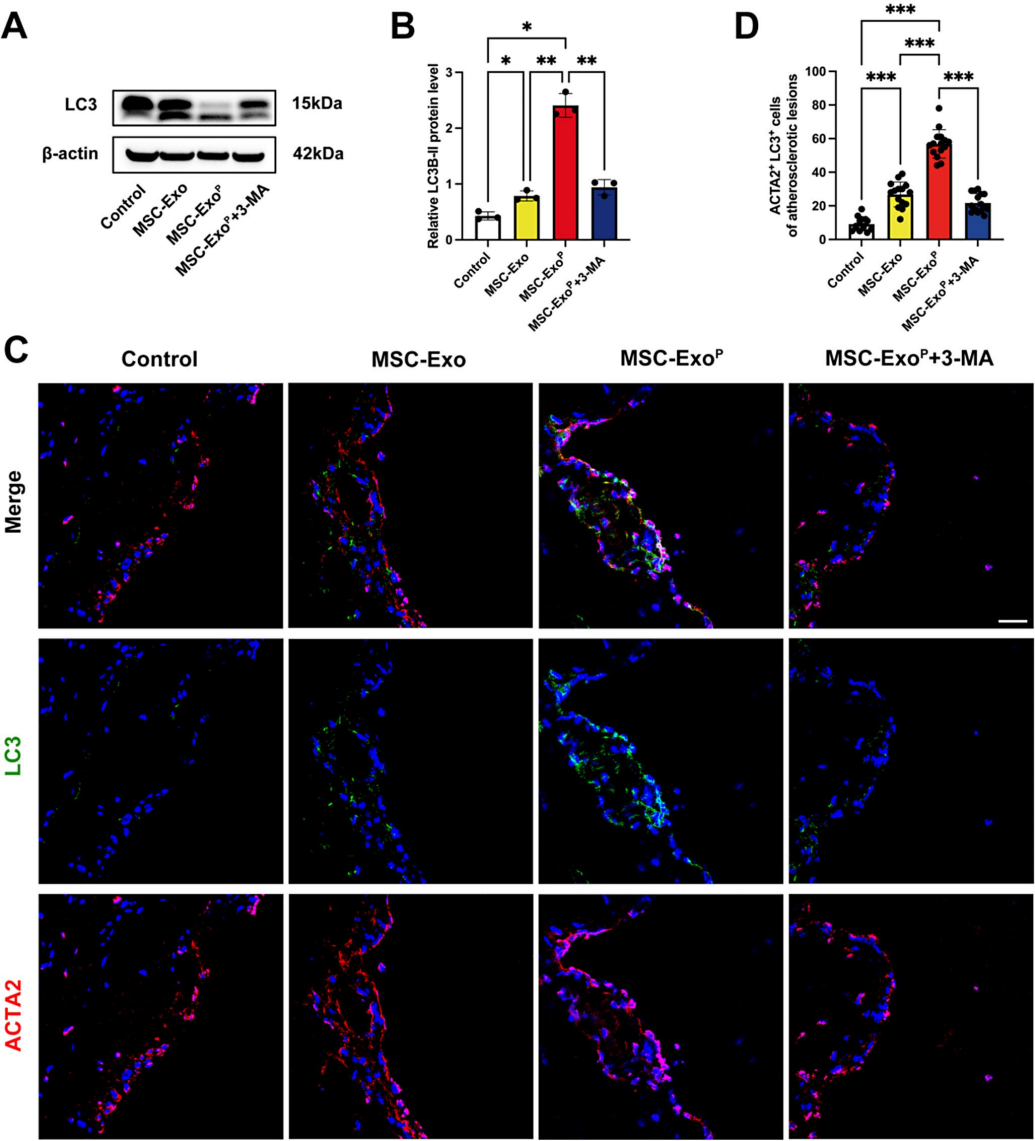
Activation of VSMC autophagy in vivo. (**A**) Western blot analysis and (**B**) quantification of LC3-II/ILC3-I after 4-week treatment with PBS, MSC-Exo, MSC-Exo^P^ or MSC-Exo^P^+3-MA in vivo. *n* = 3. (**C**) Representative confocal images of LC3-ACAT2 in aortic root sections of different treatment groups. (red: ACTA2, green: LC3, blue: nuclei) Scale bar = 25 μm. *n* = 14–15. (**D**) Quantification of the number of LC3^+^ACTA2^+^ cells of atherosclerotic plaques. All data are presented as mean ± SD (*P < 0.05, **P < 0.01, ***P < 0.001)

To further explore whether 4-weeks treatment of MSC-Exo^P^ on ApoE^−/−^ mice promoted VSMC autophagy, we first detected the expression of LC3-II/LC3-I ratio. The MSC-Exo^P^ group displayed a higher level of LC3-II/LC3-I ratio, compared with MSC-Exo group (*P* < 0.01) and control group (*P* < 0.05), and the increase in LC3-II/LC3-I ratio was reversed by 3-MA treatment (*P* < 0.01) (Fig. 7A and B). Besides, we analyzed the autophagy-related markers LC3 and smooth muscle cell marker ACTA2 located within plaques in the aortic root of the atherosclerosis model using immunofluorescence staining (Fig. 7C and D). Our confocal microscopy analysis found that under MSC-Exo^P^ conditions, the number of LC3^+^ACTA2^+^ cells increased significantly relative to MSC-Exo group (*P* < 0.001) and control group (*P* < 0.001). Moreover, the effect of the MSC-Exo^P^ on the activation of VSMC autophagy was also blocked in mice treated with 3-MA (*P* < 0.001). Taken together, these data demonstrated that MSC-Exo^P^ had a superior targeting capability for atherosclerotic plaques and strengthened VSMC autophagy in atherosclerotic lesions.

### Inhibition of VSMC proliferation, migration, and foam cell formation by inducing autophagy in vivo

**Fig. 8:**
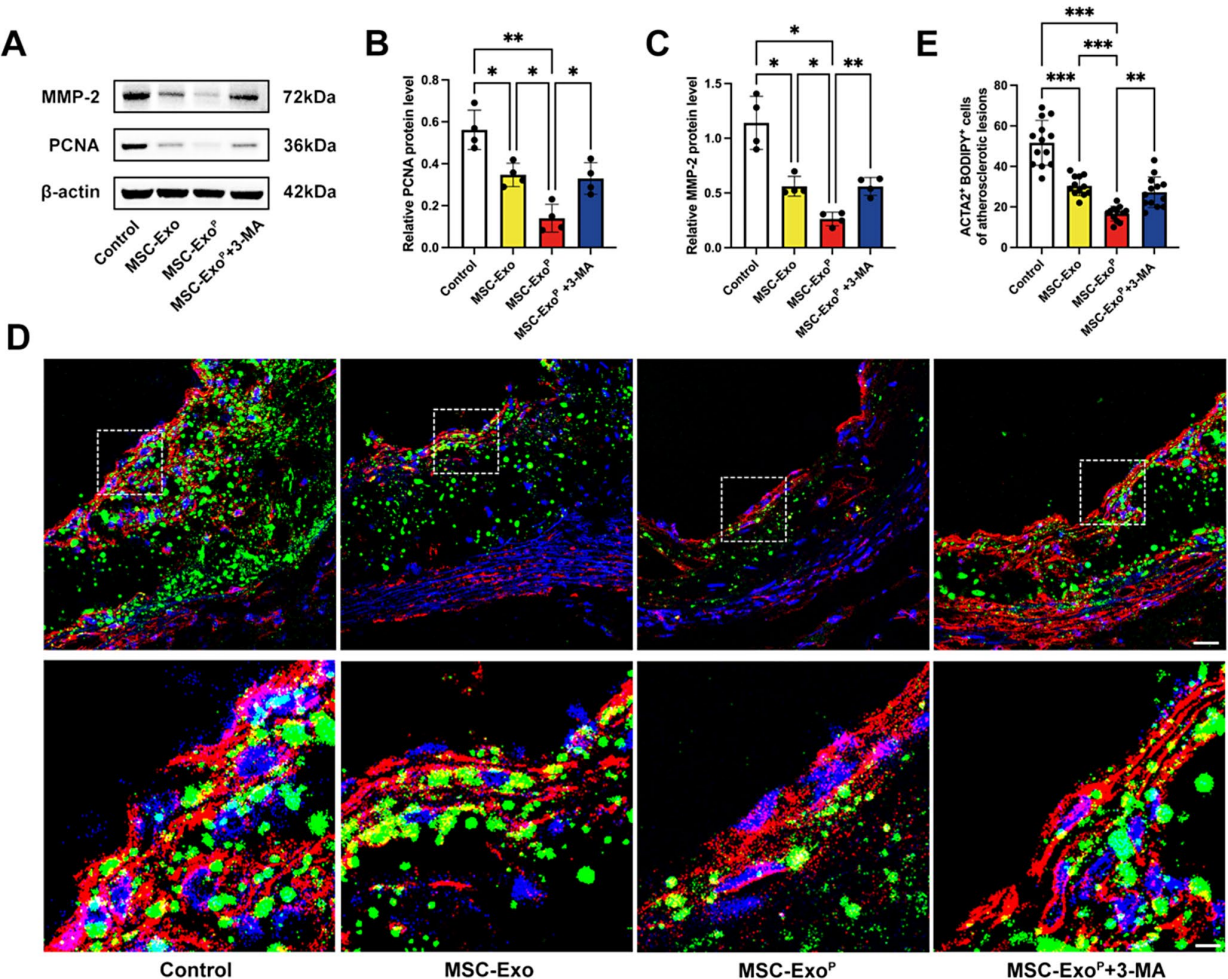
Inhibition of VSMC proliferation, migration, and foam cell formation in vivo. (**A**) Western blot analysis and (**B**-**C**) quantification of PCNA and MMP-2 after 4-week treatment with PBS, MSC-Exo, MSC-Exo^P^ or MSC-Exo^P^+3-MA in vivo. *n* = 4. (**D**) Representative confocal images of ACAT2-BODIPY in aortic root sections of different treatment groups. (red: ACTA2, green: BODIPY, blue: nuclei) Scale bar = 25 μm and scale bar = 5 μm (Enlarge). *n* = 12–13. (**E**) Quantification of the number of ACTA2^+^BODIPY^+^ cells atherosclerotic plaques. All data are presented as mean ± SD (*P < 0.05, **P < 0.01, ***P < 0.001)

Considering the indispensable role of VSMCs in the development of atherosclerosis, we first explored the direct effect of MSC-Exo^P^ on VSMC proliferation and migration in vivo. As shown by western blot (Fig. 8A-C), the expression level of PCNA in MSC-Exo^P^ group was significantly decreased compared with MSC-Exo group (*P* < 0.05) and control group (*P* < 0.01). Additionally, the MMP-2 protein level was also decreased after the treatment of MSC-Exo^P^ when compared with other groups (both *P* < 0.05), and the therapeutic effect of MSC-Exo^P^ was reversed by 3-MA both in VSMC proliferation and migration (both *P* < 0.05). To further identify the direct effect of MSC-Exo^P^ on VSMC-derived foam cell formation in vivo, we then stained the plaques in the aortic root with ACTA2 and BODIPY (Fig. 8D and E). As expected, the number of ACTA2^+^ BODIPY^+^ cells was decreased in MSC-Exo^P^ group compared with other groups (both *P* < 0.001). Moreover, this decrease was hindered by 3-MA treatment (*P* < 0.01), indicating that MSC-Exo^P^ reduced lipid accumulation and the number of VSMC-derived foam cells by inducing VSMC autophagy. Together, these results indicated that MSC-Exo^P^ were of excellent therapeutic efficiency in inhibition of VSMC proliferation, migration, and foam cell formation by activating autophagy better than MSC-Exo.

### Biosafety assessment

**Fig. 9:**
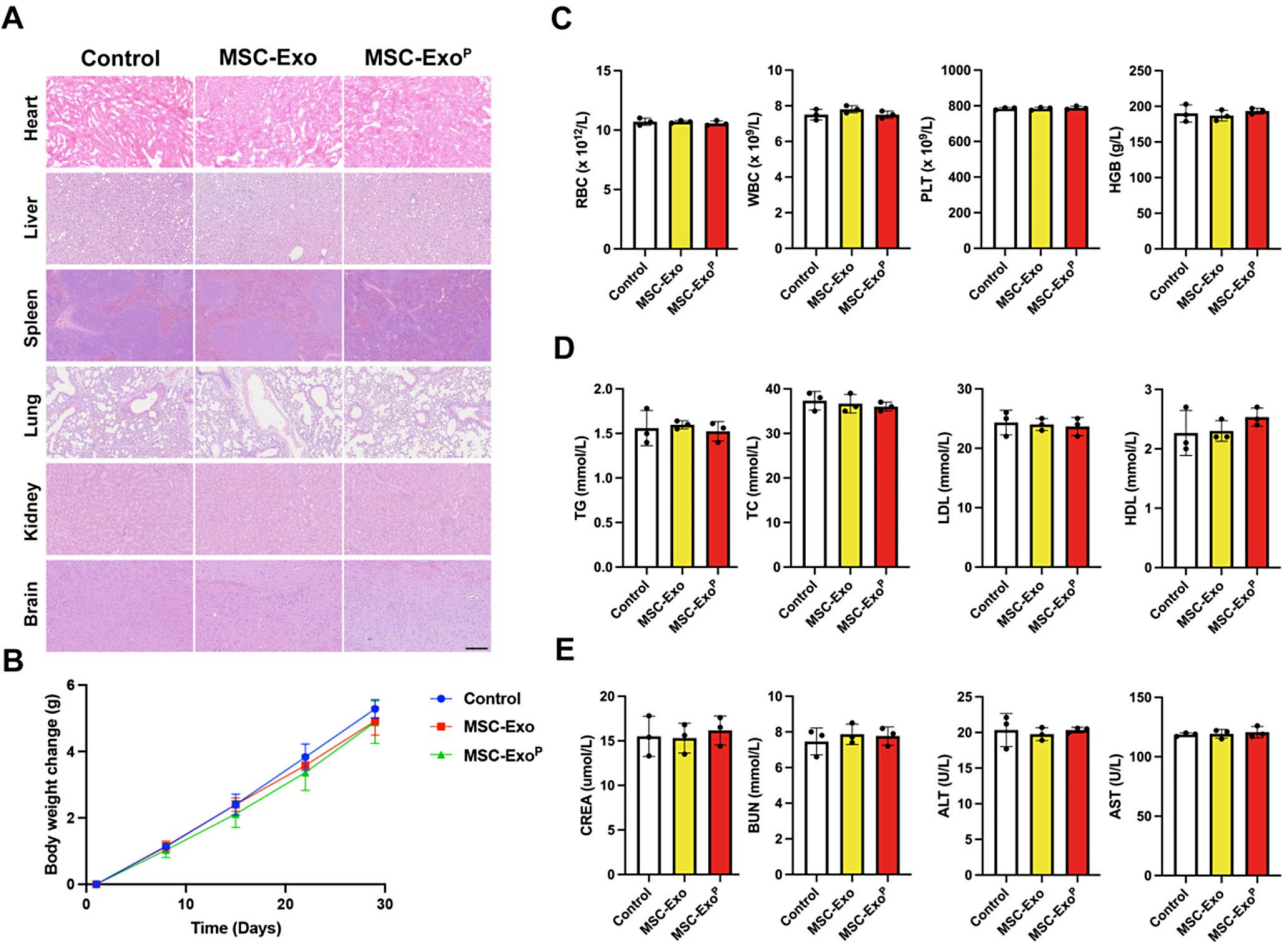
Biosafety assessment of MSC-Exo^P^. (**A**) Representative H&E images of major organs of ApoE^−/−^ mice after 4-week treatment with PBS, MSC-Exo or MSC-Exo^P^ in vivo. Scale bar = 200 μm. (**B**) The changes in mice body weight after 4-week different treatments. *n* = 6. (**C**) Quantitative analysis of complete blood count after long-term various treatments. *n* = 3. (**D**) Quantitative analysis of the level of serum lipids in different treatment groups. *n* = 3. (**E**) Quantitative analysis of CREA, BUN, ALT, and AST in various treatment groups. *n* = 3. All data are presented as mean ± SD

To further evaluate the in vivo biosafety of MSC-Exo^P^, mice were injected with MSC-Exo^P^ or MSC-Exo for 4 w, and then tissue histology, complete blood count, and blood biochemistry analysis were performed. H&E staining of major organs showed no obvious abnormalities or injuries in MSC-Exo^P^ group and MSC-Exo group compared with PBS group (Fig. 9A), and the body weight of the mice showed a normal increase trend without significant difference after various treatment (Fig. 9B). Complete blood count results of red blood cells (RBC), white blood cells (WBC), platelets (PLT), and hemoglobin (HGB) were in normal ranges in various groups (Fig. 9C). There were no obvious differences in serum lipids levels, including triglyceride (TG), total cholesterol (TC), low-density lipoprotein (LDL), and high-density lipoprotein (HDL) (Fig. 9D). Besides, the levels of creatinine (CREA), blood urea nitrogen (BUN), alanine aminotransferase (ALT), and aspartate aminotransferase (AST) in various treatment groups showed no distinguishable differences, indicating that treatment with MSC-Exo^P^ had little effect on hepatic and kidney functions (Fig. 9E). These results implicated that MSC-Exo^P^ had good biocompatibility and no remarkable negative effect and toxicity in vivo.

## Discussion

In this study, we developed platelet membrane-fused exosomes for targeted treatment of atherosclerosis, leveraging their natural homing ability to atherosclerotic plaques. Our findings validated that MSC-Exo^P^ biomimetic drug-delivery system exhibited remarkable specificity in targeting atherosclerotic plaques and enhanced the internalization of MSC-Exo^P^ by VSMCs. Both in vitro and in vivo results demonstrated that MSC-Exo^P^ could induce VSMC autophagy, which subsequently inhibited VSMC proliferation, migration, and foam cell formation. This mechanism played an important role in slowing the progression of atherosclerotic lesions without causing major organ toxicity or adverse reactions.

Exosomes derived from MSCs play an important role in the treatment of cardiovascular diseases, particularly atherosclerosis. Although MSCs have therapeutic potential in alleviating atherosclerosis, such as reducing inflammation, improving dyslipidemia, and stabilizing vulnerable plaques [27, 28], allogeneic stem cell transplantation may trigger immune rejection in the host and may lead to abnormal proliferation after transplantation, which increases the risk of tumor formation. Exosomes, being smaller in size, reduce the risk of local blockage in the microvascular system and improve delivery efficiency [29, 30]. Unlike single drugs or siRNA therapies, which usually act on a specific target without multi-pathway regulation [31–33], exosomes carry a variety of active molecules from their parent stem cells, including miRNAs, proteins, and lipids, allowing them to play multiple regulatory roles in target cells and achieve overall improvement of atherosclerotic lesions [30].

The initiation and progression of atherosclerosis often begin with arterial endothelial dysfunction. MSC-derived exosomes can effectively deliver miR-145 to endothelial cells (ECs), downregulating JAM-A expression, inhibiting EC migration, and reducing atherosclerotic plaque formation [34]. Additionally, adipose-derived MSC exosomes (ADSC-Exo) alleviate EC apoptosis by inhibiting the mitochondrial-dependent apoptosis signaling pathway [35]. MSC-derived exosomes also regulate the vascular immune environment by reducing excessive macrophage infiltration through inhibition of the IGF2BP1/PTEN pathway [36]. miRNAs such as miR-let7 and miR-21a-5p in MSC-derived exosomes downregulate NF-κB and KLF6 signaling pathways, inhibiting proinflammatory factor release, shifting macrophages towards an anti-inflammatory M2 phenotype, and thereby reducing inflammatory damage [36, 37]. In addition to regulating macrophage polarization, MCS-Exos also reduce inflammation by inhibiting Nod-like receptor protein 3 (NLRP3) expression to decrease macrophage pyroptosis [38, 39]. MSC-Exo can transfer miR-125b to VSMCs, downregulating Myosin-1E (Myo1e), a gene associated with VSMC proliferation and migration, thus reducing atherosclerotic plaque formation [25]. Moreover, MSC-derived exosomes mitigate oxidative stress by reducing reactive oxygen species (ROS) production [40] and activate the PPARγ/LXRα/ABCA1 signaling pathway to inhibit intracellular lipid accumulation [41], collectively highlighting the promising therapeutic potential of exosomes in treating atherosclerosis.

Although MSC-derived exosomes show good application prospects in the treatment of atherosclerosis, the therapeutic effect is limited due to the very low retention rate and targeting ability of exosomes in local atherosclerotic lesions. To increase the targeting ability and therapeutic effects of exosomes, various strategies have been explored in the field of engineering exosomes up to date, such as genetic engineering, chemical modification, and membrane fusion techniques. Through exosome genetic engineering technology, targeting ligands or homing peptides could be expressed on the exosome membrane surface. However, the high cost and unstable plasmid transfection efficiency required further exploration of gene engineering technology [42]. Alternatively, exosomal surfaces could be directly inserted with different compounds via chemical modification, including covalent and non-covalent modification [43]. Through chemical modification to deliver active exosomes into the local atherosclerotic lesions, the targeting ability and therapeutic effects were greatly enhanced [44]. Nonetheless, neither genetic engineering nor chemical modification technique could circumvent the need for a single targeting motif selection and the insufficient simulation of complex multicellular interaction characteristic of atherosclerosis further limited their application.

Compared with genetic engineering and chemical modification technique, membrane fusion technique attracted greater attention. In this study, we employed membrane fusion technology to fabricate a plaque-targeting delivery system by fusing MSC-Exo with natural platelet membranes. MSC-Exo^P^ was well coated with platelet membranes, preserving its cup-shaped nanostructures, and displaying a comparable range to that of MSC-Exo. The targeting ability of the platelet membrane is not limited to a single binding of a specific ligand-receptor, but also contains a variety of adhesion molecules, which can realize the recognition of multiple targets in plaques. When an endothelial injury occurs, glycoproteins such as GPIbα, GPIIb/IIIa, and integrins express on the surface of platelets and promote adhesion between platelets and impaired endothelial cells by binding to its corresponding ligand [45]. Compared with MSC-Exo, an enhanced fluorescent intensity of atherosclerotic aortas and aortic roots after incubating with DiI-labeled MSC-Exo^P^ in vitro or intravenously injecting DiI-labeled MSC-Exo^P^ was observed. Membrane fusion technology also offers significant advantages by simplifying the production process, reducing toxicity risks and enhancing biological stability, making it a promising approach for targeted delivery research. In addition to improving plaque targeting, previous studies have shown that activated platelets could be internalized by VSMCs within hours to days under conditions of inflammation [46]. The expression of ICAM-1 by VSMCs was greatly upregulated [47], which could mediate platelet adhesion by interacting with platelet αIIbβ3 [48, 49]. In our study, we confirmed that the expression of ICAM-1 by VSMCs was increased significantly upon exposure to ox-LDL stimulation. Subsequently, we incubated VSMCs to MSC-Exo^P^ labeled with DiI and observed a conspicuously stronger fluorescence intensity in the cytoplasm, suggestive of the potential enhancement of VSMC uptake efficiency facilitated by platelet membrane fusion.

Autophagy, a vital cellular metabolic process, could break down and degrade protein aggregates and abnormal organelles [50]. Targeted activation of autophagy-related pathways has emerged as a novel strategy for preventing and treating atherosclerosis. In atherosclerotic disease, autophagy activation involves multiple critical molecular pathways and mechanisms. In this context, Adenosine monophosphate-activated protein kinase (AMPK) plays a protective role by activating autophagy through inhibition of the mammalian target of rapamycin (mTOR) pathway, which typically suppresses autophagy. AMPK activation reduces mTOR activity, thereby initiating autophagy, decreasing macrophage proliferation and inflammation [51, 52], and enhancing endothelial cell survival under hypoxia and oxidative stress to maintain vascular integrity [53]. Additionally, pathways such as PI3K/Akt, MAPK, and GSK-3 also contribute to autophagy enhancement by inhibiting mTOR, which further limits VSMC proliferation, inflammation, and oxidative damage, stabilizing vulnerable plaques [54–56]. Oxidative stress can accumulate ROS, activating AMPK and p53 pathways to further initiate autophagy and autophagosome formation [57]. These pathways intersect, forming a complex autophagy regulatory network that plays a protective role in atherosclerotic lesions.

Although previous studies have examined the regulatory effects of various pathways on autophagy, the role of MSC-derived exosomes in modulating autophagy in atherosclerosis remains unexplored. Our study is the first to investigate the regulatory impact of exosomes on autophagy in atherosclerosis, particularly their role in activating autophagy in VSMCs. During the early stage of atherosclerosis, the proliferation and migration of VSMCs under the intima lead to the formation of plaques and the stenosis of vascular lumens. Along with the proliferation and migration process, the release of cytokines and inflammatory factors incurs damage to the structure of vascular wall [6, 58]. Moreover, studies have revealed that the primary source of foam cells in atherosclerotic plaques stems from VSMCs, thereby reinforcing their critical role in the development of atherosclerosis [5]. Our study demonstrated that pre-stimulation of VSMCs with ox-LDL resulted in impairment of VSMC autophagy, which was subsequently reversed upon administration of MSC-Exo^P^. The activation of VSMC autophagy was further confirmed by an increased LC3-II/LC3-I ratio and number of LC3^+^ACTA2^+^ cells within atherosclerotic plaques after intravenous injection of MSC-Exo^P^. These findings suggested that the MSC-Exo^P^ exhibited greater efficacy in activating VSMC autophagy both in vitro and in vivo. Previous studies have primarily focused on the role of exosomes in inflammation regulation and cell communication. This study is the first to demonstrate that exosomes can also regulate the autophagy process in VSMCs, filling a crucial gap in atherosclerosis treatment regarding the role of exosomes in autophagy modulation. Moreover, by utilizing platelet membrane fusion technology, we significantly enhanced the plaque-targeting and uptake efficiency of exosomes, thereby strengthening their effect on autophagy activation. We further examined the impact of exosome-mediated autophagy activation on VSMC phenotype. Both in vivo and in vitro experiments showed that autophagy activation inhibited VSMC proliferation, migration, and foam cell formation, thereby slowing the progression of atherosclerosis. Additionally, the targeted delivery of platelet membrane-fused exosomes markedly amplified the regulatory effects of autophagy on VSMC phenotypic shifts, offering a more efficient approach that prevented coronary artery restenosis, reduced necrotic core volume, and improved plaque stability. This innovative exosome-targeted delivery method provides new insights and strategies for the precise treatment of atherosclerosis.

Our study has several limitations. First, given the critical role of VSMCs in atherosclerosis progression, the present study primarily focused on the regulatory effects of exosomes on these cells. However, the effects on other cell types within atherosclerotic plaques, such as endothelial cells, macrophages, and immune cells, warrant further investigation. Second, the main purpose of this study was to evaluate and validate the feasibility and therapeutic potential of platelet membrane-fused exosomes for targeted treatment of atherosclerosis. While we confirmed the autophagy-activating effects of exosomes in VSMCs, the specific molecular pathways and underlying mechanisms were not explored in depth. Third, the long-term safety of the platelet membrane-fused exosome delivery system and its potential effects on the systemic immune system were not thoroughly assessed in this study.

## Conclusion

In summary, we successfully developed a novel atherosclerosis-targeting drug delivery system utilizing platelet membrane modified exosomes. Our investigation unveiled that the improvement of targeting ability and the activation of VSMC autophagy by MSC-Exo^P^ conferred benefits in suppressing the advancement of atherosclerosis. The MSC-Exo^P^ biomimetic drug delivery system appeared as the new safe and effective drug targeting delivery system and presented a promising future for clinical translation in the treatment of atherosclerotic cardiovascular diseases and even for other diseases. Meanwhile, we should also extend this approach to membranes of other cell types or load other anti-atherosclerotic biomolecules for targeting atherosclerosis treatment.

## Data Availability

The datasets used and/or analyzed during the current study are available from the corresponding author upon reasonable request.
